# Demultiplexing-free ultra-compact WDM-compatible multimode optical switch assisted by mode exchanger

**DOI:** 10.1515/nanoph-2024-0201

**Published:** 2024-07-04

**Authors:** Siwei Liu, Xin Fu, Jiaqi Niu, Yujie Huo, Chuang Cheng, Lin Yang

**Affiliations:** Key Laboratory of Optoelectronic Materials and Devices, Institute of Semiconductors, Chinese Academy of Sciences, P.O. Box 912, Beijing 100083, China; Center of Materials Science and Optoelectronics Engineering, University of Chinese Academy of Sciences, Beijing 100049, China

**Keywords:** mode-division multiplexing, optical switch, on-chip optical interconnects, silicon photonics

## Abstract

Silicon-based optical switches are integral to on-chip optical interconnects, and mode-division multiplexing (MDM) technology has enabled modes to function as carriers in routing, further boosting optical switches’ link capacity. However, traditional multimode optical switches, which typically use Mach–Zehnder interferometer (MZI) structures and mode (de)multiplexers, are complex and occupy significant physical space. In this paper, we propose and experimentally demonstrate a novel demultiplexing-free dual-mode 3 × 3 thermal-optical switch based on micro-rings (MRs) and mode exchangers (MEs). All MRs are designed to handle TE_1_ mode, while the ME converts TE_0_ mode to TE_1_ mode, enabling separate routing of both modes. Bezier curves are employed to optimize not only the ME, but also the dual-mode 45° and 90° waveguide bends, which facilitate the flexible and compact layout design. Moreover, our structure can support multiple wavelength channels and spacings by adding pairs of MRs, exhibiting strong WDM compatibility. The switch has an ultra-compact footprint of 0.87 × 0.52 mm^2^. Under both “all-bar” and “all-cross” configurations, its insertion losses (ILs) remain below 8.7 dB at 1,551 nm, with optical signal-to-noise ratios (OSNRs) exceeding 13.0 dB. Also, 32 Gbps data transmission experiments validate the switch’s high-speed transmission capability.

## 1 Introduction

With the ever-increasing demand for computational speed and transmission capacity in supercomputers and data centers, traditional electrical interconnects are facing significant challenges in terms of bandwidth and power dissipation [1], [2]. To overcome these issues, optical interconnects with large bandwidth, low power consumption, low latency, and multiplexing compatibility are extensively studied and deemed promising for applications across various distance scenarios [3], [4]. Benefiting from wavelength-division multiplexing (WDM) technology, the transmission capacity of optical interconnects has been elevated to a higher level [5], [6]. However, its dependence on multiple laser sources and limited communication bandwidth have further sparked interest in another space-division multiplexing (SDM) technique. Based on multiple cores or orthogonal modes in fibers or waveguides, these parallel space channels offer a new means to avoid capacity crunch beyond WDM [7], [8], [9]. In particular, monolithically integrated mode-division multiplexing (MDM) systems on the silicon-on-insulator (SOI) platform, leveraging their advantage of complementary metal-oxide-semiconductor (CMOS) compatibility and high refractive index contrast, hold great promise in high-density on-chip optical interconnects among multiprocessors [10], [11]. Recently, various silicon-based multimode devices have been reported, such as mode multiplexers [12], [13], multimode waveguide crossings [14], [15], multimode waveguide bends [16], [17], and multimode power splitters [18], [19].

Reconfigurable optical switches, which enable flexible data flow reallocation and enhance information throughput, are pivotal components for optical interconnects [20]. Over the past few decades, significant breakthroughs have been achieved in on-chip silicon-based optical switches. Many high-performance single-mode optical switches have been proposed, with some reaching scales as large as 240 × 240 [21], [22], [23], [24], [25], [26]. Recent advancements in MDM technology have propelled research in multimode optical switches, and two routing schemes have been demonstrated [27], [28], [29], [30], [31], [32], [33], [34], [35]. One scheme is to process multimode signals simultaneously using a mode-insensitive Mach–Zehnder optical switch. For example, a mode-insensitive 2 × 2 three-mode optical switch is demonstrated in Ref. [27], based on a pair of multimode interference (MMI) 3 dB couplers and a widened phase shifter. However, the components are relatively large, and this scheme fails to achieve varied routing functions for distinct mode channels. The other scheme is to demultiplex multimode signals into single-mode signals, which are then handled separately by single-mode optical switches. In this way, general architectures for on-chip multimode optical switches are discussed in Ref. [28], and a global 2 × 2 four-mode optical switch is demonstrated based on Mach–Zehnder interferometer (MZI) structures. Although the demultiplexing-switching-multiplexing process facilitates the global exchange of space and mode, it introduces additional steps and significantly increases the total footprint.

Compared to broadband MZI structures, narrowband micro-ring (MR) structures naturally possess wavelength selectivity and can efficiently reduce device size and power consumption. In Ref. [30], an MR-based dual-mode optical switch with two wavelength channels is demonstrated, but it is still constrained by the complex (de)multiplexing operations, and the 1 × 2 port structure is not efficient compared to a double-input-port structure. Based on a hybrid MZI-MR structure, a demultiplexing-free 2 × 2 dual-mode optical switch is proposed in Ref. [29]. However, the introduction of MZI increases the total footprint, and to be compatible with WDM, the spacing of its adjacent wavelength channels is solely determined by the MR’s free spectral range (FSR), which is susceptible to fabrication errors and challenging to meet the diverse needs of MDM-WDM hybrid systems.

In this paper, we design and experimentally demonstrate an ultra-compact WDM-compatible dual-mode 3 × 3 optical switch on a standard SOI platform. Unlike conventional schemes, we introduce trajectory-optimized mode exchangers (MEs) to achieve a demultiplexing-free structure. Based on the Spanke-Benes architecture, the switch comprises three 2 × 2 optical switch units (OSUs), with each OSU consisting of a pair of MRs, a waveguide crossing, and four MEs. All MRs handle TE_1_ mode, while TE_0_ mode is exchanged to TE_1_ mode for processing. To make the layout more flexible and compact, we employ Bezier curves to optimize dual-mode 45° and 90° waveguide bends, facilitating smooth connections between the crossings and MRs. The total footprint is only 0.87 × 0.52 mm^2^. Since pairs of MRs can be easily added in each OSU, the wavelength channels can be further expanded to accommodate various channel spacings and quantities, which exhibit robust WDM compatibility. Experimental results in both “all-bar” and “all-cross” states show insertion losses (ILs) below 8.7 dB and optical signal-to-noise ratios (OSNRs) exceeding 13.0 dB at 1,551 nm. The switching time of the device is 24.8 μs. Besides, all optical links exhibit a 3 dB signal bandwidth greater than 0.32 nm, and transmission experiments using 32 Gbps OOK signals demonstrate the switch’s high-speed transmission capability.

The remainder of this article is organized as follows: Section 2 describes our dual-mode optical switch’s principle and building blocks. Section 3 presents the experimental results of the OSU and the 3 × 3 switch network. In Section 4, we discuss the switch’s scalability, including wavelength scalability and mode scalability. Finally, we conclude in Section 5.

## 2 Design of the device

### 2.1 Working principle of the switch

**Figure 1:**
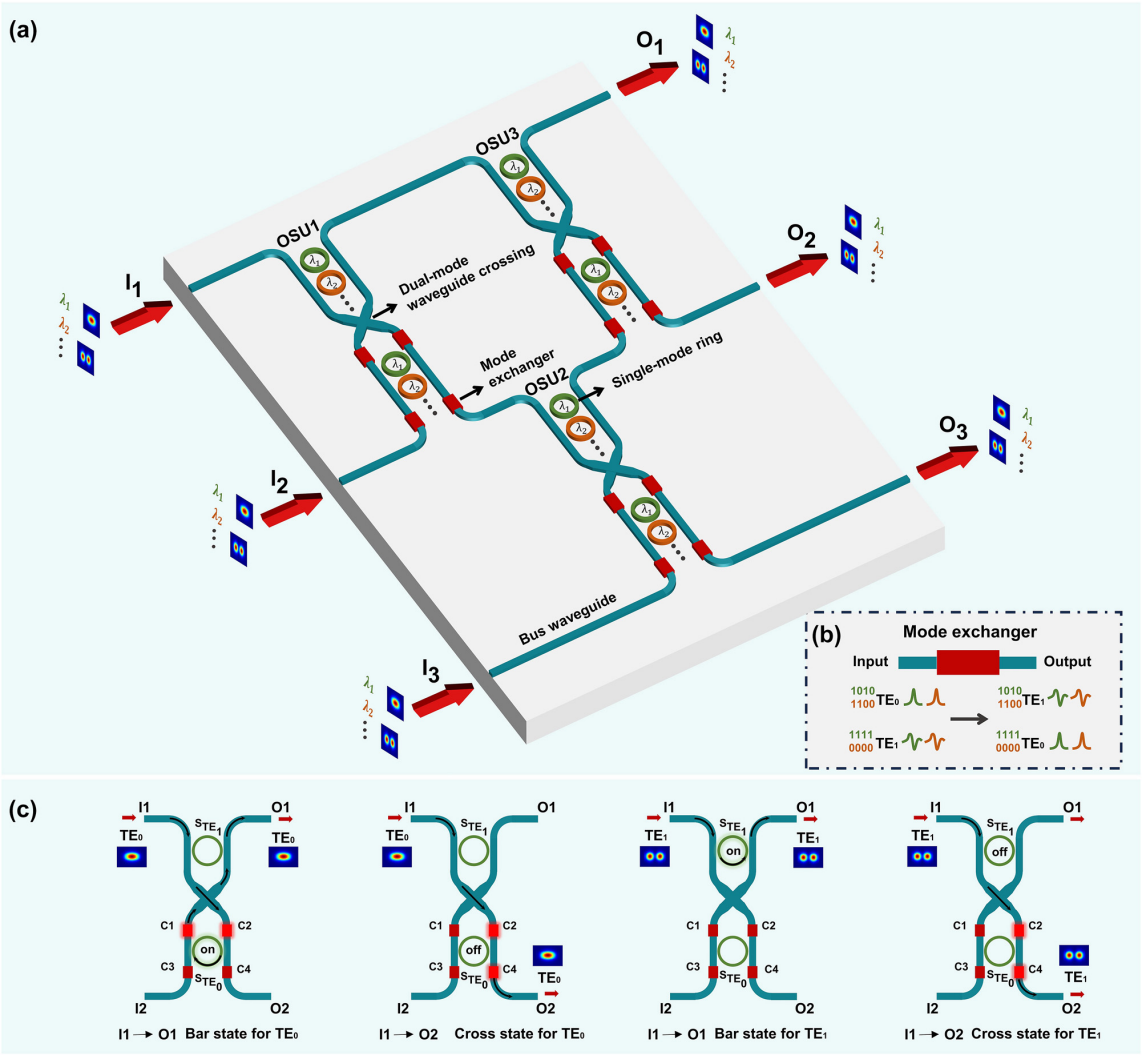
The proposed dual-mode 3 × 3 optical switch. (A) The main schematic. (B) Working principle of the ME. (C) The four possible scenarios when TE_0_ or TE_1_ mode is incident from the input port I1 of the OSU.

Figure 1A illustrates the schematic of the 3 × 3 dual-mode optical switch based on the Spanke-Benes architecture. This architecture includes three 2 × 2 OSUs, and each OSU comprises a waveguide crossing, four MEs, several waveguide bends, and *N* pairs of add-drop MRs, where *N* represents the number of configured wavelength channels. Each pair of MRs, distinguished by different colors, operates in its assigned wavelength channel, with one MR routing TE_0_ mode and the other routing TE_1_ mode. The blue bus waveguides support TE_0_ and TE_1_ modes simultaneously, while all rings only support the fundamental mode TE_0_. In the coupling region of the MR, TE_0_ mode inside the ring matches TE_1_ mode within the bus waveguide. Consequently, in each OSU, multimode signals first pass through MRs that operate for TE_1_ mode. Subsequently, as shown in Figure 1B, the ME swaps the mode indices, enabling manipulation of the information carried by TE_0_ mode through an equal number of MRs. To achieve reconfigurable control, each MR integrates a heater on top, switching between ON and OFF states by adjusting the DC voltage applied to the heater. In the ON state, the resonant wavelength of the MR aligns with the operating wavelength, directing the signal through the ring to the drop port. Conversely, in the OFF state, the resonant wavelength is adjusted away from the operating wavelength, routing the optical signal directly to the through port.

For clarity of presentation, we use a single-wavelength OSU to illustrate the scenarios when an optical signal is incident from one input port I1, as shown in Figure 1C. The routing states for TE_0_ and TE_1_ modes are determined by the ON and OFF states of the two MRs denoted as S_TE0_ and S_TE1_, respectively. Within the OSU, there are four MEs denoted as C1 – C4. When S_TE0_ is ON, TE_0_ mode follows a path through C2, S_TE0_, C1, and finally reaches the output port O1 (I1 – O1, bar state for TE_0_). Conversely, when S_TE0_ is OFF, TE_0_ mode passes through C2 and C4, propagating to the output port O2 (I1 – O2, cross state for TE_0_). Similarly, when S_TE1_ is ON, TE_1_ mode travels into S_TE1_ and transmits from the drop port to the output port O1 (I1 – O1, bar state for TE_1_). When S_TE1_ is OFF, TE_1_ mode passes through C2 and C4, reaching the output port O2 (I1 – O2, cross state for TE_1_). All paths go through an even number of MEs, ensuring consistency of the mode order before and after the OSU. Since the routing control of TE_0_ and TE_1_ modes is independent, the OSU can not only achieve path exchange with mode insensitivity but also enable the recombination of modes from different inputs. Thus, there are 2! × 2! = 4 routing states for the single-wavelength OSU. Correspondingly, the 3 × 3 dual-mode optical switch has 3! × 3! = 36 routing states. As the switch network expands to *N* × *N* ports, the number of routing states escalates to *N*! × *N*!.

### 2.2 Design of the building blocks

The design and considerations for the building blocks of the OSU are explained as follows.

**Figure 2:**
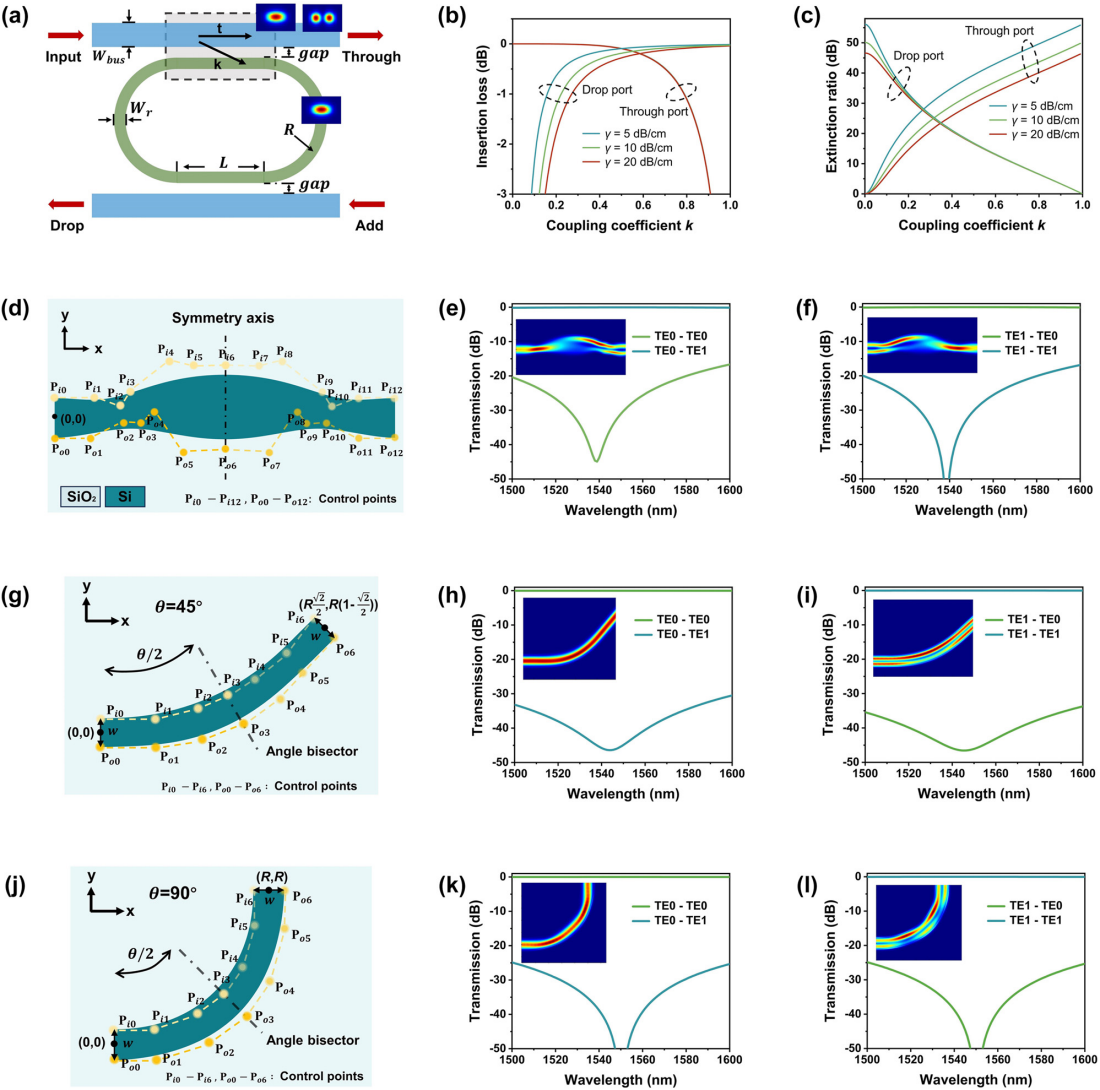
Design of the main components in the OSU. (A) Scattering matrix model of the add-drop MR. The calculated results of the (B) IL and (C) ER at both the through and drop ports with different *k* and *γ*, respectively. (D) The schematic of the ME. The transmission spectra of the ME for (E) TE_0_ and (F) TE_1_ modes. (G) The schematic of the dual-mode 45° waveguide bend. The transmission spectra of the 45° bend for (H) TE_0_ and (I) TE_1_ modes. (J) The schematic of the dual-mode 90° waveguide bend. The transmission spectra of the 90° bend for (K) TE_0_ and (L) TE_1_ modes.

Firstly, we model the add-drop MR using the scattering matrix and adopt symmetric coupling for port balance, as shown in Figure 2A. With the self-coupling coefficient *t* and cross-coupling coefficient *k* in the coupling region, the transmittance of the through port *I*_through_ and the drop port *I*_drop_ are derived as follows,

(1) $$I_{\text{through}}=\frac{t^{2}+\alpha^{2}t^{2}-2\alpha t^{2}\cos\theta }{1+\alpha^{2}t^{4}-2\alpha t^{2}\cos\theta },$$

(2) $$I_{\text{drop}}=\frac{\alpha k^{4}}{1+\alpha^{2}t^{4}-2\alpha t^{2}\cos\theta },$$

where *α* is the round-trip amplitude attenuation factor, and *θ* is the round-trip phase shift induced by the circulating optical signal. We assume lossless coupling (i.e., *t* ^2^ + *k* ^2^ = 1) for simplification. To trade off bending loss against FSR, we choose the ring’s radius as 7 μm. The transmission attenuation *γ* in the ring cavity is estimated to be within 5–20 dB/cm, depending on the certain fabrication level. Based on Equations (1) and (2), the insertion loss (IL) and extinction ratio (ER) at the through and drop ports with different *k* and *γ* are calculated and illustrated in Figure 2B and C, respectively. It can be observed that both the IL and ER exhibit an opposite tendency for the through and drop ports as *k* increases. The cross-coupling coefficient *k* is chosen to be around 0.36 to balance the performance at both ports. Considering single-mode conditions within the ring and phase matching in the coupling region, the width of the bus waveguide *W*_bus_ and the width of the ring *W*_*r*_ are fixed as 0.9 μm and 0.43 μm, respectively. For robustness against fabrication errors, the coupling gap is fixed at 210 nm. Then, to achieve the desired coupling strength (*k* ∼ 0.36) around the 1,550 nm operating wavelength, the ring is designed as a racetrack type with a track length *L* of 5 μm.

Secondly, the dual-mode ME is optimized using two independent Bezier curves. As shown in Figure 2D, the ME is horizontally symmetrical along the centerline, with the upper and lower boundaries defined by the Bezier curves. The waveguide widths at the starting and ending positions are set to 1.4 μm, while the length is preset to 12 μm. Leveraging the adjoint gradient method integrated into the Lumopt toolbox (Ansys Lumerical), we optimize the positions of the Bezier curves’ control points through the 3D finite-difference-time-domain (FDTD) solver. We started the optimization with lower-order Bezier curves and successfully optimized the ME using 12th-order Bezier curves. Taking the waveguide center of the starting position as the coordinate origin, the coordinates of the control points on the left side of the symmetry axis are specified as follows (all in μm): $P_{i0}\left(0,0.7\right)$ , $P_{i1}\left(1.43,0.7\right)$ , $P_{i2}\left(2.29,0.3\right)$ , $P_{i3}\left(2.63,0.78\right)$ , $P_{i4}\left(4,1.92\right)$ , $P_{i5}\left(4.88,1.5\right)$ , $P_{i6}\left(6,1.7\right)$ , $P_{o0}\left(0,-0.7\right)$ , $P_{o1}\left(1.22,-0.7\right)$ , $P_{o2}\left(2.4,-0.21\right)$ , $P_{o3}\left(3.1,-0.29\right)$ , $P_{o4}\left(3.46,0.23\right)$ , $P_{o5}\left(4.53,-1.33\right)$ , and $P_{i6}\left(6,-1.11\right)$ . The simulated results depicted in Figure 2E and F shows ILs < 0.04 dB and crosstalk (CT) <−31 dB at 1,550 nm. Over the broad wavelength range of 1,500–1,600 nm, the CT remains below −17 dB.

Thirdly, we discuss the design of dual-mode waveguide routing devices, including waveguide crossings and bends. As for the dual-mode waveguide crossing, we adopt the structure proposed in Ref. [36], which combines fully etched and shallowly etched waveguides in the MMI coupling region to achieve the overlap of self-imaging positions for TE_0_ and TE_1_ modes. In order to make the layout of the switch more flexible, we conducted trajectory optimization to create 45° and 90° dual-mode waveguide bends. As shown in Figure 2G and J, both bends are symmetric along the bisector of corresponding angles and determined by two independent Bezier curves (inner and outer boundaries). The effective radii *R* for 45° and 90° bends are preset as 10 μm and 5 μm, respectively, and the waveguide widths *w* are both fixed as 1 μm at their starting and ending positions. Similarly, we optimized the coordinates of the control points using the adjoint gradient method and successfully optimized the bends using 6th-order Bezier curves. For the 45-degree bend, these points are specified as follows (all in μm): $P_{i0}\left(0,0.5\right)$ , $P_{i1}\left(0.84,0.5\right)$ , $P_{i2}\left(1.69,0.54\right)$ , $P_{i3}\left(3.91,0.56\right)$ , $P_{o0}\left(0,-0.5\right)$ , $P_{o1}\left(0.94,-0.50\right)$ , $P_{o2}\left(1.86,-0.33\right)$ , and $P_{o3}\left(4.53,-0.94\right)$ . For the 90-degree bend, the points are specified as follows (all in μm): $P_{i0}\left(0,0.5\right)$ , $P_{i1}\left(0.8,0.5\right)$ , $P_{i2}\left(1.87,0.6\right)$ , $P_{i3}\left(3.94,1.06\right)$ , $P_{o0}\left(0,-0.5\right)$ , $P_{o1}\left(0.98,-0.5\right)$ , $P_{o2}\left(2.52,-0.21\right)$ , and $P_{o3}\left(4.8,0.2\right)$ . The simulation results are shown in Figure 2H and I for the 45-degree bend, as well as in Figure 2K and L for the 90-degree bend. For both bends, the CT remains below −25 dB within the wavelength range of 1,500–1,600 nm.

### 2.3 Design fabrication

**Figure 3:**
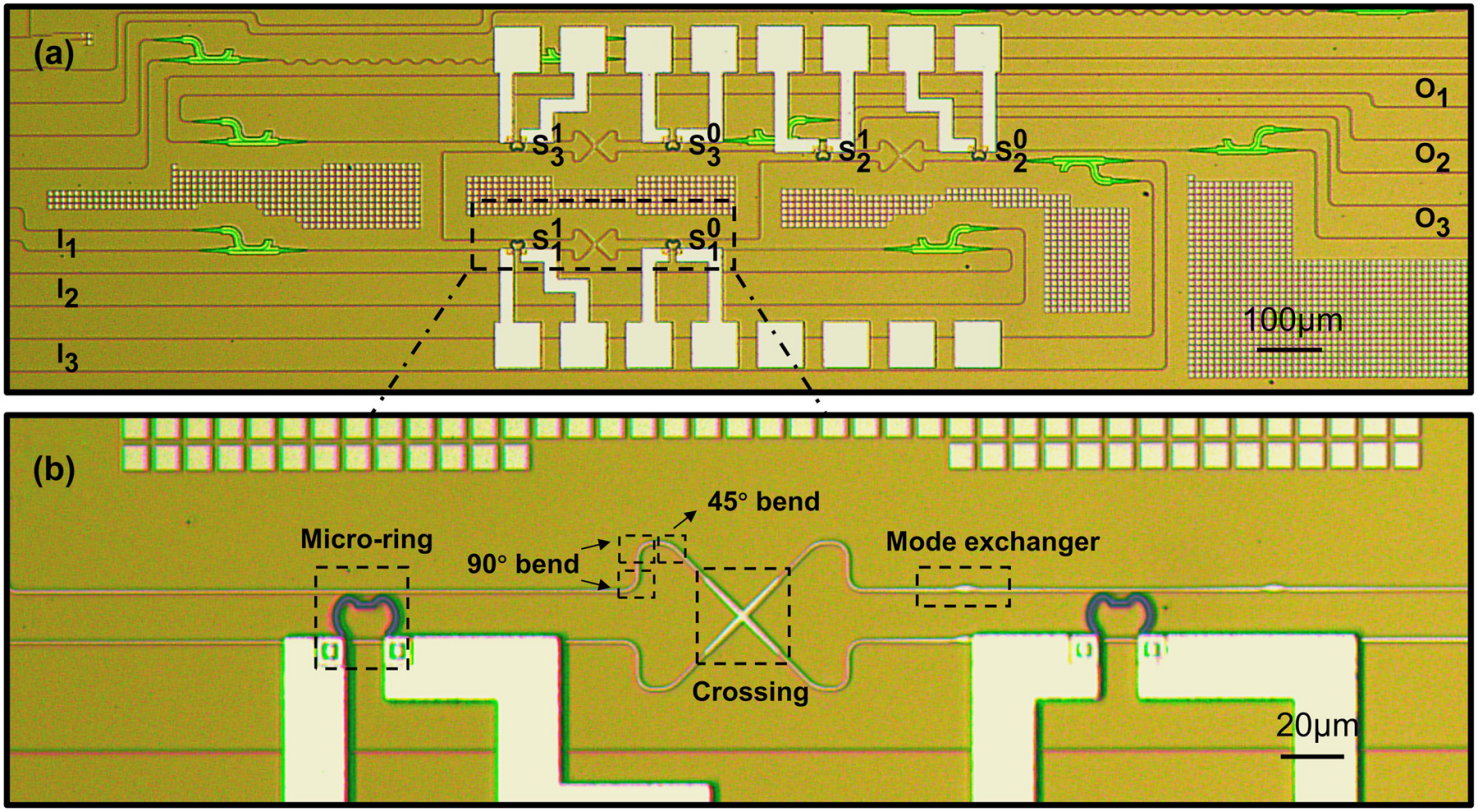
Optical microscope images of (A) the dual-mode 3 × 3 thermal-optical switch and (B) the zoomed-in view of one OSU.

For a clear and simple proof of concept, we configure a single wavelength channel, and the dual-mode 3 × 3 optical switch is fabricated on a multi-project wafer (MPW) with a 220 nm-thick top silicon layer and a 3 μm-thick buried silicon dioxide layer at Advanced Micro Foundry, Singapore. It takes a footprint of 0.87 × 0.52 mm^2^. To generate and characterize dual-mode signals, we integrate mode multiplexers and demultiplexers based on asymmetry directional couplers at the input ports (*I*_1_, *I*_2_, *I*_3_) and output ports (*O*_1_, *O*_2_, *O*_3_). The micrograph of the fabricated device is shown in Figure 3, with each MR labeled as $S_{i}^{m}\left(i=1,2,3;m=0,1\right)$ , where *i* corresponds to the index of the OSU consistent with Figure 1A and *m* is the mode index.

## 3 Experiment and result analysis

### 3.1 Switching element characterization

Firstly, we characterize the performance of the passive components. The experimental configuration is as follows: light launched from the broadband amplified spontaneous emission (ASE) is coupled into the chip through an optical fiber and is then directed into an optical spectrum analyzer (OSA) to monitor the spectra. When measuring crosstalk, we test the spectrum of a single device. To obtain accurate IL results, we measure the spectrum of multiple cascaded devices, normalize it, and take the average. Here, we present the test results at 1,550 nm in the following. For the ME, the ILs for TE_0_ and TE_1_ modes are 0.026 dB and 0.021 dB, respectively, and the CT is less than −22.5 dB. For the crossing, the ILs for TE_0_ and TE_1_ modes are 1.52 dB and 1.81 dB, respectively, and the CT is less than −21.0 dB. For the 90° bend, the ILs for TE_0_ and TE_1_ modes are 0.010 dB and 0.009 dB, respectively, and the CT is less than −20.9 dB. For the 45° bend, the ILs for TE_0_ and TE_1_ modes are 0.004 dB and 0.013 dB, respectively, and the CT is less than −21.8 dB. The large loss observed in the dual-mode waveguide crossing deviates from Ref. [36], which is speculated to stem from the fabrication sensitivity of the MMI coupling region. More stable etching precision is desired to reduce the ILs. The CT values of the above devices are worse than the simulated values, attributed to fabrication errors and limitations imposed by the mode multiplexer used for the test.

**Figure 4:**
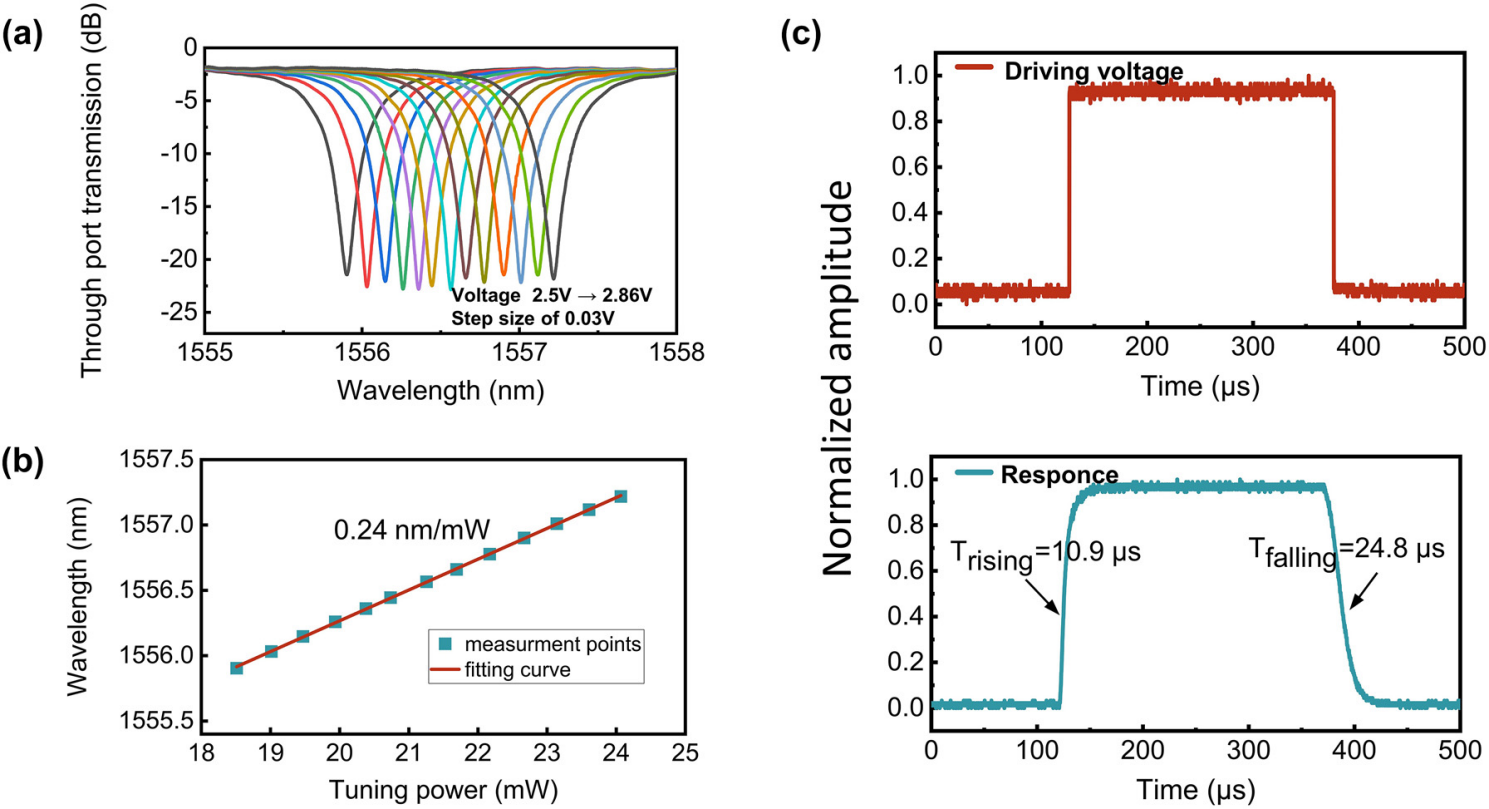
The thermal-optic and time-domain responses of the OSU. (A) The MR’s through-port transmission under thermal bias ranges from 2.5 V to 2.86 V in a 0.03 V step size. (B) The linear fit of the resonant wavelength and the tuning power. (C) The normalized square wave driving signal (upper) and corresponding dynamic response (lower).

Next, we measure the thermal-optic and time-domain responses of the OSU. Due to the consistent behavior of the two modes at the MRs, we selectively measure the thermal-optic response of S_TE0_. By adjusting the DC voltage applied to S_TE0_, we measure the spectrum of the I1 – O2 link (refer to the port labels in Figure 1C). As shown in Figure 4A, the resonant wavelength experiences a redshift as the DC voltage changes from 2.5 V to 2.86 V. In Figure 4B, linear fitting of the resonant wavelength and tunning power yields a thermal tuning efficiency of 0.24 nm/mW or 30 GHz/mW, corresponding to a *P*_*π*_ of about 21 mW. Subsequently, we measure the switch reconfiguration times by analyzing the time-domain response. The ON and OFF states for S_TE0_ (bar and cross states for TE_0_ mode) are toggled with 2 kHz square wave electrical signals, and a digital communication analyzer (DCA) is used to record the dynamic response time of the output optical signals. As shown in Figure 4C, the 10 %–90 % rising and falling times are 10.9 μs and 24.8 μs, respectively.

### 3.2 Transmission spectra of the dual-mode 3 × 3 optical switch

By adjusting the drive voltage applied to the three OSUs, our dual-mode 3 × 3 switch can freely switch among 36 routing states. Here, we sequentially set the two modes of all OSUs to the “bar” and “cross” states and refer to them as “all-bar” and “all-cross” states to demonstrate the device’s transmission characteristics. We choose 1,551 nm as the operating wavelength, and the resonance wavelength of each MR differs by FSR/2 in the cross and bar states.

**Figure 5:**
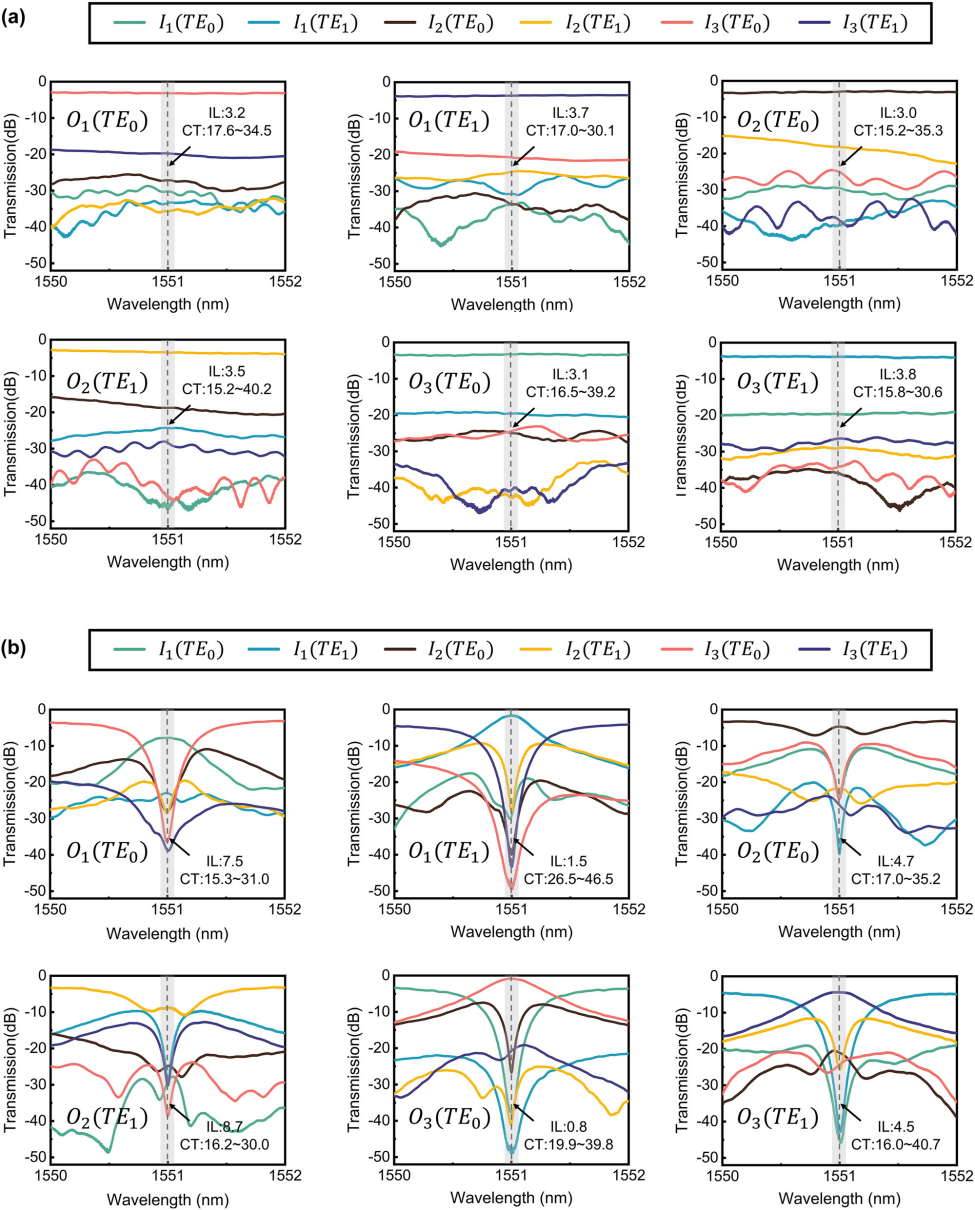
The normalized transmission spectra of the dual-mode 3 × 3 optical switch in (A) the ‘‘all-cross’’ state and (B) the “all-bar” state.

The measured spectra in the “all-cross” state are depicted in Figure 5A, where each plot illustrates the transmission spectra of each output port from all six input ports, and the ILs of the edge couplers and mode (de)multiplexers have been subtracted. The ILs of TE_0_ mode channels range from 3.0 to 3.2 dB, while those of TE_1_ mode channels range from 3.5 to 3.8 dB. All signal links do not enter the ring; instead, they sequentially pass through four MEs, two waveguide crossings, and several waveguide bends. The discrepancy in ILs between TE_0_ and TE_1_ mode channels is primarily from the waveguide crossings. For the optical noise at each output port, the inter-mode CT is larger than the inter-path CT, attributed to the cumulative effects of dual-mode waveguide crossings, dual-mode waveguide bends, MEs, and the mode (de)multiplexers used for measurement. The maximum inter-mode CT is 15.2 dB, and the worst optical signal-to-noise ratio (OSNR) is 13.8 dB by summing the crosstalk.

We then toggle the dual-mode 3 × 3 optical switch to the “all-bar” state and show the spectra in Figure 5B. Unlike the “all-cross” state, the ILs in the “all-bar” state exhibit significant fluctuations from 0.8 dB to 8.7 dB, attributed to the different numbers of waveguide crossings in each signal link. Specifically, the link $I_{3}\left(\text{T}{\text{E}}_{0}\right)-O_{3}\left(\text{T}{\text{E}}_{0}\right)$ with an IL of 0.8 dB avoids all crossings, while the link $I_{2}\left(\text{T}{\text{E}}_{1}\right)-O_{2}\left(\text{T}{\text{E}}_{1}\right)$ with an IL of 8.7 dB passes through the maximum number of crossings (4 times). Fabrication errors significantly deteriorated the performance of the crossing, leading to a notable increase in the overall ILs and loss inconsistency. Using dual-ring MRs instead of single-ring MRs can help avoid crossings in the OSU. For all signal links, the 3 dB bandwidth exceeds 0.32 nm (40 GHz), and the worst-case CT is 15.3 dB, with the corresponding worst-case OSNR being 13.0 dB.

As listed in Table 1, the power consumptions for the “all-cross” and “all-bar” states are 23.5 mW and 139.9 mW, respectively. Fabrication errors lead to inconsistent initial resonant wavelengths, and there is a large tuning range between each MR’s two states (*P*_*π*_). To mitigate the power consumption, we can optimize the shape of thermal electrodes [37] or incorporate thermal isolation trenches [38] into the MRs in the future.

**Table 1:** Driving voltages and power consumptions of all MRs under the “all-cross” and “all-bar” states.

OSU		$S_{1}^{0}$	$S_{1}^{1}$	$S_{2}^{0}$	$S_{2}^{1}$	$S_{3}^{0}$	$S_{3}^{1}$
“all-bar”	Voltage (V)	1.02	1.37	0	0.75	1.16	1.6
	Current (mA)	4	5	0	2	4	4
	Power (mW)	4.08	6.85	0	1.5	4.64	6.4
“all-cross”	Voltage (V)	2.68	2.76	2.5	2.65	2.69	2.84
	Current (mA)	9	9	8	8	9	9
	Power (mW)	24.12	24.84	20	21.2	24.21	25.56

### 3.3 High-speed transmission

**Figure 6:**
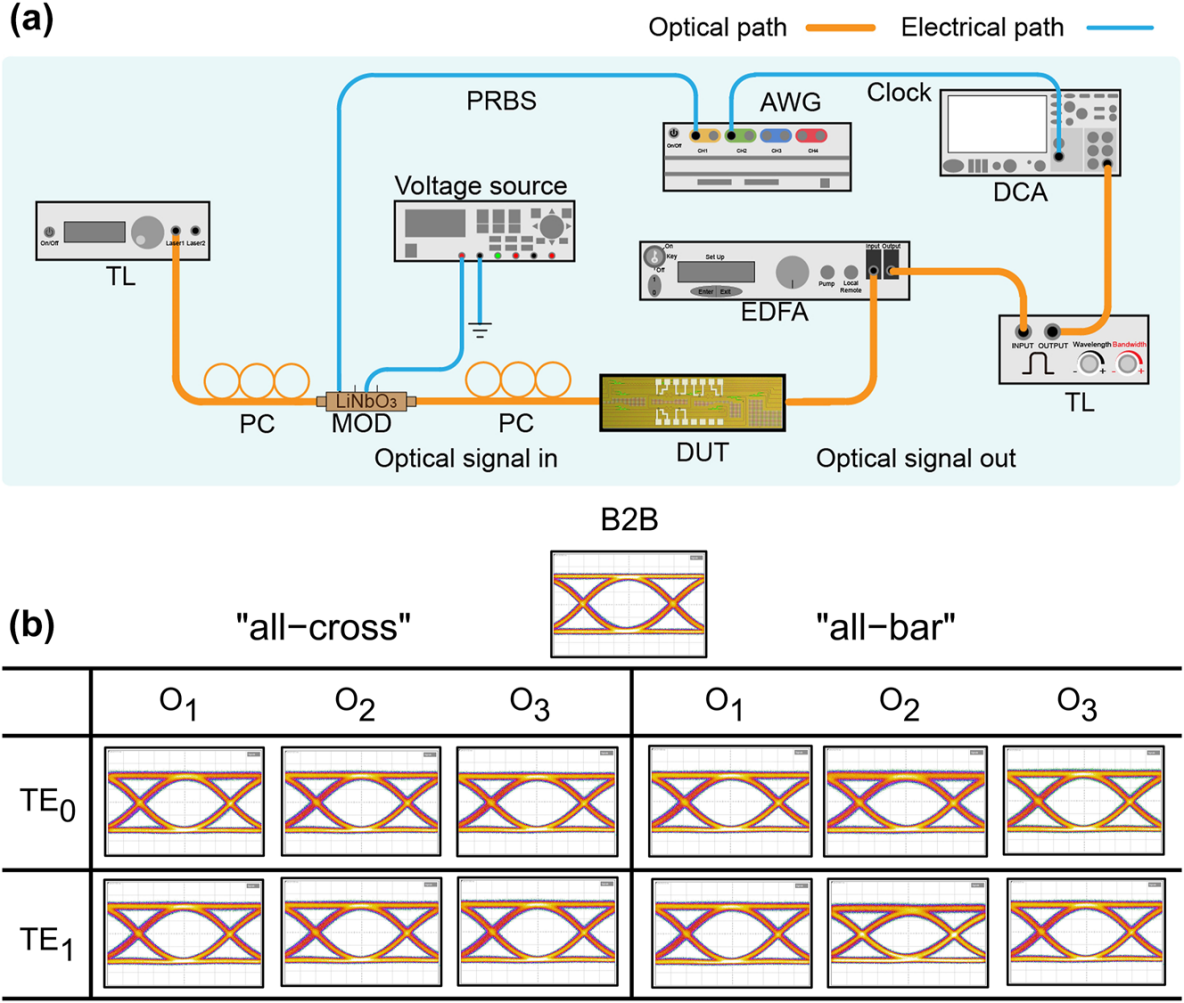
The high speed transmission experiment. (A) Experimental setup for characterizing the eye diagram of the 3 × 3 optical switch (TL, tunable laser; MOD, modulator; PC, polarization controller; AWG, arbitrary waveform generator; DUT, the device under test; EDFA, erbium-doped fiber amplifier; DCA, digital communication analyzer; OSA, optical spectrum analyzer). (B) Eye diagrams for 32 Gb/s data transmission at 1,551 nm.

The experimental setup for data transmission is depicted in Figure 6A. A tunable laser (TL) emits a continuous-wave laser at 1,551 nm, then modulated by a commercial lithium niobate modulator driven by a 32 Gbps non-return-to-zero (NRZ) signal. After polarization adjustment into the transverse electric (TE) polarization by the polarization controller (PC), the signal enters the device under test (DUT). Upon exiting the chip, the processed optical signal is amplified by an erbium-doped fiber amplifier (EDFA), filtered by a tunable filter, and finally sent to the digital communication analyzer (DCA) to record the eye diagram. We perform the 32 Gbps transmission experiments for all links in the “all-cross” and “all-bar” states. Besides, the back-to-back (B2B) eye diagram, which bypasses the DUT, is recorded directly for comparison. As shown in Figure 6B, all eye diagrams are clear and open, with slight degradation caused by the ILs of the device and the noise from the EDFA. Despite the relatively non-uniform link losses, these results indicate that our switch can exchange 32 Gbps NRZ optical signals with dual-mode high signal integrity.

## 4 Scalability discussion

### 4.1 Wavelength scalability

**Figure 7:**
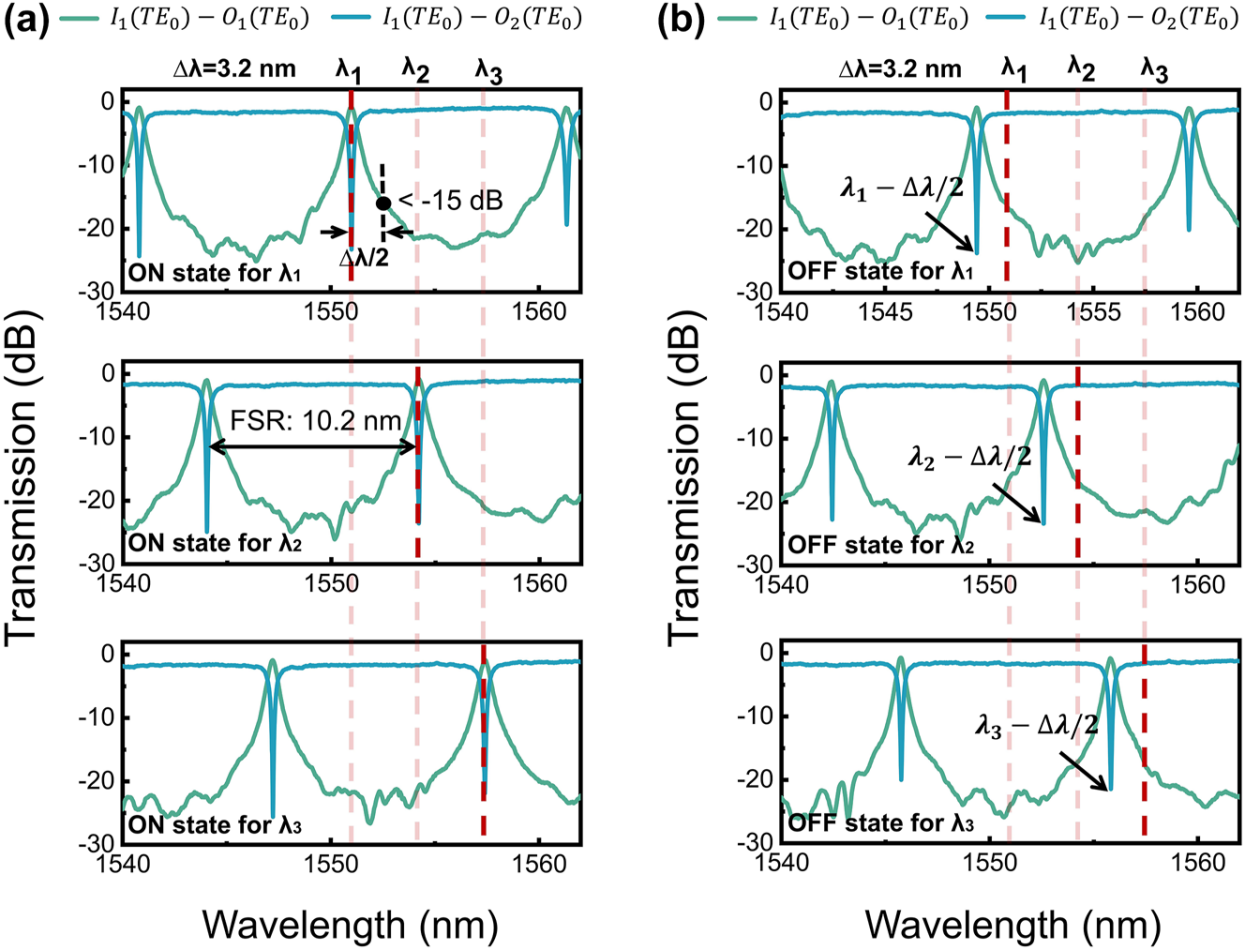
The illustration of wavelength scalability. The spectra of TE_0_ mode from the input port I1 of the OSU when the operating wavelength is adjusted to *λ*_1_, *λ*_2_, and *λ*_3_ in (A) the ON (bar) state and (B) the OFF (cross) state.

As mentioned in Section 2.1, the wavelength channels can be expanded by configuring different numbers of MR pairs. Here, leveraging the wavelength tuning of a single OSU, we delve into a discussion on the switch’s wavelength scalability. To simulate the case of multiple wavelength channels, we set the wavelength spacing Δ*λ* to 3.2 nm (∼400 GHz) and sequentially adjust the operating wavelength to *λ*_1_ (1,551 nm), *λ*_2_ (1,554.2 nm) and *λ*_3_ (1,557.4 nm). Taking TE_0_ mode launched from the input port I1 as an example, Figure 7A and B illustrate the spectra of TE_0_ mode in the bar (ON) and cross (OFF) states, respectively. Unlike the single-wavelength scenario, the spacing of resonant wavelengths for the MR’s ON and OFF states in each wavelength channel is set to Δ*λ*/2. Thus, for the wavelength of *λ*_*i*_ ± Δ*λ*/2(*i* = 1, 2, 3), the transmittance at the MR’s drop port should be low enough, and the transmittance at the through port should be high enough to achieve low channel crosstalk. An MR with a larger roll-off rate is desired to suppress the crosstalk further and achieve smaller channel spacing. However, the drop port spectrum of a single-ring MR is essentially a Lorentzian curve with a low shape factor (roll-off rate), which makes it challenging to balance between a large bandwidth and a large roll-off rate. We will consider using OSUs composed of cascaded dual-ring MRs to address this problem, like the structure presented in Ref. [39]. Additionally, due to the wavelength dependency of the components, the resonance peak shapes vary at different wavelengths. When configuring multiple wavelength channels with a considerable interval between the minimum and maximum wavelengths, using different MR parameters for different wavelengths helps reduce channel non-uniformity.

The FSR determines the range to accommodate wavelength channels. Once Δ*λ* is selected, the maximum number of wavelength channels is *N* = FSR/Δ*λ*. To obtain a larger FSR, we can shorten the ring’s circumference by reducing the coupling gap or the ring’s radius. As proposed in Ref. [40], the modified Bezier curves can help reduce the bend radius to around 1 μm, leading to an FSR exceeding 20 nm. Additionally, it is worth noting that the signals from *N* wavelength channels can independently be in the bar or cross state to achieve wavelength-selective function, which exhibits strong routing flexibility.

### 4.2 Mode scalability

**Figure 8:**
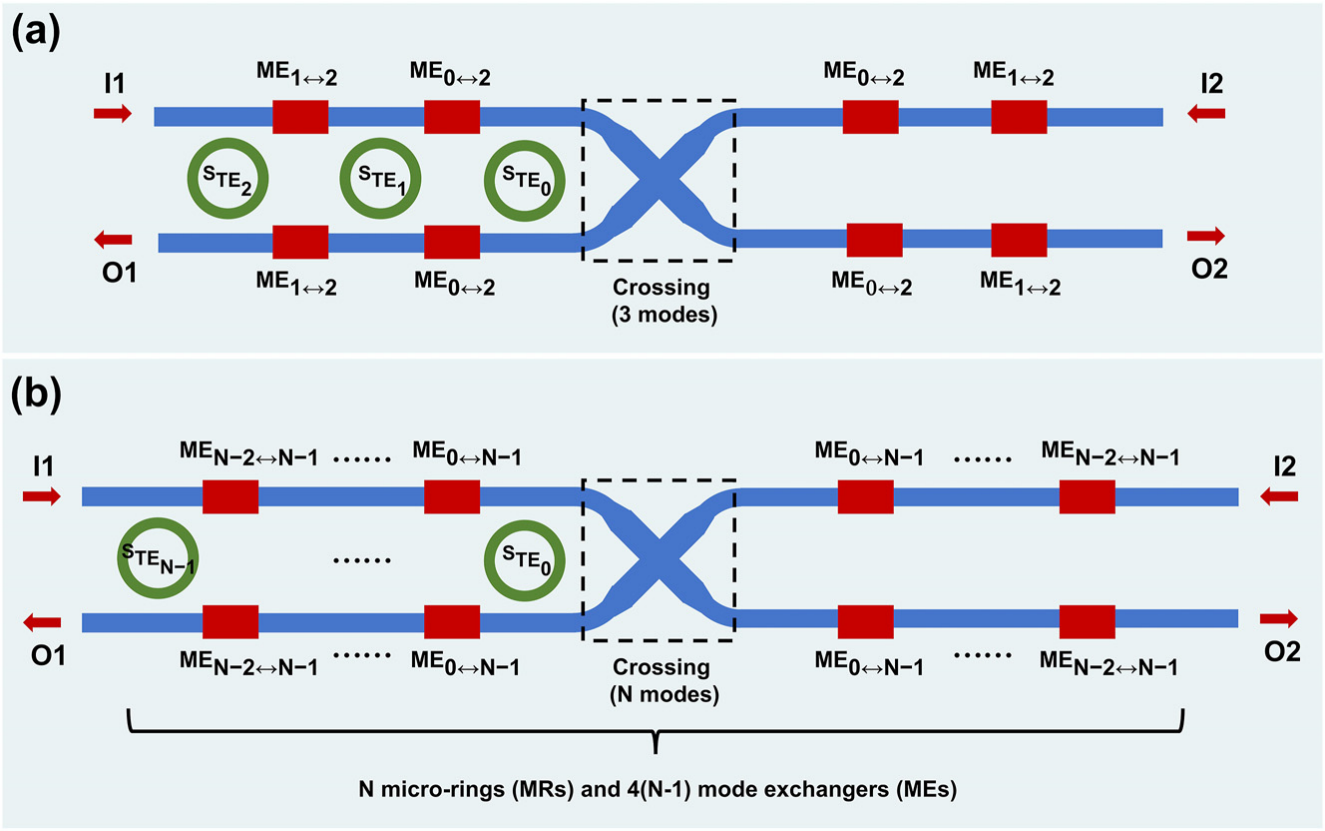
The illustration of mode scalability. The schematic diagrams of the expanded OSUs that can accommodate (A) 3 modes and (B) *N* modes.

To demonstrate mode scalability, we schematically depict the single-wavelength OSU extended to 3 modes and *N* (≥3) modes. As shown in Figure 8A, the 3-mode 2 × 2 OSU is composed of one three-mode waveguide crossing, four MEs for TE_1_ and TE_2_ modes ME_1↔2_, four MEs for TE_0_ and TE_2_ modes ME_0↔2_, and three MRs. The blue bus waveguides support all three modes, while the green rings only support the fundamental mode. Within the coupling region of the MR, TE_2_ mode within the bus waveguide matches TE_0_ mode inside the ring. Selecting the highest-order mode to match the fundamental mode in the ring is intended to ensure that the ring width is sufficiently small to avoid exciting higher-order modes. Therefore, TE_2_ mode is first handled through S_TE2_, while TE_1_ and TE_0_ modes are sequentially converted to TE_2_ mode to be processed by S_TE1_ and S_TE0_, respectively. There is an even number of each kind of ME in all paths so that the mode index remains the same before and after the OSU. Figure 8B extends the scenario to an N-mode 2 × 2 OSU, composed of one *N*-mode waveguide crossing, 4(*N*−1) MEs, and *N* MRs. Similarly, the blue bus waveguides support all *N* modes, and within the coupling region of the MR, TE_*N*−1_ mode within the bus waveguide matches TE_0_ mode inside the ring. The 4(*N*−1) MEs are divided into (*N*−1) types, each of which is responsible for converting TE_*i*_ (*i* = 0, 1, …, *N*−2) mode into TE_*N*−1_ mode for processing while keeping other modes unchanged. Noticeably, the signals from different input ports always experience different numbers of crossings. This can also be solved by using dual-ring MRs.

The passive components mentioned here can be implemented using various methods. For example, waveguide crossings with more than two modes can be achieved using subwavelength grating [15] or the inverse design method [41]. Meanwhile, the MEs can be implemented using the inverse design method [42] or the trajectory optimization method as presented in Section 2.2.

## 5 Conclusions

In conclusion, we demonstrate an ultra-compact dual-mode 3 × 3 thermal-optical switch based on MR structure. The switch integrates MEs, enabling independent routing of the two modes while avoiding complex demultiplexing and re-multiplexing operations. Experimental characterization in both “all-cross” and “all-bar” states shows ILs less than 8.7 dB and OSNRs larger than 13.0 dB at 1,551 nm. Additionally, 32 Gbps data transmission experiments verify the high-speed transmission capability. We also discuss the feasibility of extending the OSU to more wavelength and mode channels. Overall, the demonstrated dual-mode 3 × 3 silicon optical switch holds promise for advancing high-density WDM-MDM hybrid optical networks. Also, our design ideas and methods may inspire the development of other optical interconnect devices.
